# Adolescents’ use and perceived usefulness of generative AI for schoolwork: exploring their relationships with executive functioning and academic achievement

**DOI:** 10.3389/frai.2024.1415782

**Published:** 2024-08-28

**Authors:** Johan Klarin, Eva Hoff, Adam Larsson, Daiva Daukantaitė

**Affiliations:** Department of Psychology, Lund University, Lund, Sweden

**Keywords:** generative AI, executive functions, learning, cognition, academic achievement, ChatGPT

## Abstract

In this study, we aimed to explore the frequency of use and perceived usefulness of LLM generative AI chatbots (e.g., ChatGPT) for schoolwork, particularly in relation to adolescents’ executive functioning (EF), which includes critical cognitive processes like planning, inhibition, and cognitive flexibility essential for academic success. Two studies were conducted, encompassing both younger (Study 1: *N* = 385, 46% girls, mean age 14 years) and older (Study 2: *N* = 359, 67% girls, mean age 17 years) adolescents, to comprehensively examine these associations across different age groups. In Study 1, approximately 14.8% of participants reported using generative AI, while in Study 2, the adoption rate among older students was 52.6%, with ChatGPT emerging as the preferred tool among adolescents in both studies. Consistently across both studies, we found that adolescents facing more EF challenges perceived generative AI as more useful for schoolwork, particularly in completing assignments. Notably, academic achievement showed no significant associations with AI usage or usefulness, as revealed in Study 1. This study represents the first exploration into how individual characteristics, such as EF, relate to the frequency and perceived usefulness of LLM generative AI chatbots for schoolwork among adolescents. Given the early stage of generative AI chatbots during the survey, future research should validate these findings and delve deeper into the utilization and integration of generative AI into educational settings. It is crucial to adopt a proactive approach to address the potential challenges and opportunities associated with these emerging technologies in education.

## 1 Introduction

After the release of ChatGPT, a Large Language Model (LLM) generative AI chatbot, a debate emerged in Sweden in spring 2023 regarding its allowance or prohibition in educational settings. At the heart of this debate were concerns about the potential for AI to be exploited for cheating, contrasted with its potential to enhance learning outcomes and promote equality in education by serving as an educational aid for students at risk of falling behind their peers. The utilization of generative AI tools in higher education is already evident, with a study in Germany revealing that two-thirds of university students employ these tools in their coursework for tasks such as text analysis, creation, problem-solving, and decision-making (von Garrel and Mayer, 2023). A similar trend is observed in Sweden, where a survey among adolescents and young adults aged 15–24 showed that 75% use generative AI for various educational purposes, including structuring presentations and papers, writing texts, studying, and social support (The Nordic Youth Barometer, 2023).^1^

These initial findings regarding AI tool usage suggest their application in strengthening behaviors associated with executive functioning (EF)—a set of cognitive processes critical for planning, concentration, and attention, encompassing working memory, inhibition, and cognitive flexibility, among others (Diamond, 2013). These functions are crucial for goal-directed behavior, especially in tackling tasks requiring significant cognitive effort. In educational settings, such goal-directed activities often involve cognitive assignments like writing papers, necessitating independent initiation, planning, and execution. Lower EFs have been linked to reduced academic achievement across various subjects and stages of life (Pino Muñoz and Arán Filippetti, 2021). Short-term effects of EF deficits can manifest as challenges in planning and problem-solving, difficulties in peer relationships, and unfinished schoolwork (Frazier et al., 2007). In the long term, the skills nurtured by EF are crucial not only for academic and social success in school but also for vital life outcomes such as employment stability and resistance to substance abuse later in life (Bailey, 2007; Miller et al., 2011).

While informative, the aforementioned surveys lack a deeper analysis of the individual conditions that may impact the use and effectiveness of generative AI, as well as the potential relationship between the use of generative AI and EF problems in adolescence. Moreover, there appears to be a scarcity of studies investigating the adoption of these tools among young adolescents aged 12–14. Therefore, in this study we aimed to provide preliminary insights into two key objectives: (1) investigating the frequency of usage and perceived usefulness of generative AI tools in schoolwork, including potential gender differences in both usage and perceived usefulness, and (2) analyzing how these patterns of usage and perceived utility are related to adolescents’ EF and academic achievement.

## 2 Literature review

### 2.1 Generative AI chatbots

Chatbots are interactive, language-based chat interfaces that automatically respond to user inquiries. They can be categorized into two groups: those utilizing pattern matching and those employing a machine learning approach. Pattern matching chatbots adhere strictly to predefined decision trees and consider only the current dialog turn. In contrast, machine learning-based chatbots can engage more flexibly with users, taking into account the entire context of a conversation (Maroengsit et al., 2019; Adamopoulou and Lefteris, 2020). Both pattern matching and machine learning-based chatbots are forms of artificial intelligence (AI), which simulates human intelligence through machines or systems (Xu et al., 2021). In the realm of chatbot applications, one historical drawback of machine learning-based AI chatbots has been the extensive amount of training data required to yield satisfactory results (Adamopoulou and Moussiades, 2020). LLMs are machine learning models designed to comprehend and generate human language text. The new generation of chatbots, powered by LLMs, exhibits remarkable capabilities due to extensive training datasets and advancements in natural language processing (NLP). These models, such as GPT-3, generate human-like text with high precision (Kasneci et al., 2023) and display emergent abilities in reasoning, planning, decision-making, and in-context learning, primarily due to their vast scale of pre-trained material (Naveed et al., 2023). However, they also carry the risk of perpetuating biases present in their training data (Bender et al., 2021). Recent evidence indicates that LLMs may propagate outdated and harmful race-based content, particularly in healthcare contexts (Omiye et al., 2023). The vast corpora of training data that enable their generative capabilities also give rise to the “black box” issue, wherein the reasoning behind AI outputs remains opaque to both users and developers (Cabitza et al., 2017). Some of these risks can be mitigated by quality filtering of data (Naveed et al., 2023) and using alignment tuning for LLMs, where human feedback is employed to make them helpful, honest, and harmless (HHH) (Askell et al., 2021). While these risks associated with bias from flawed training data and opacity of processes pose significant concerns, addressing these issues could unlock the potential of these tools to enhance cognitive processes (Baido-Anu and Ansah, 2023) and educational experiences (Al Shloul et al., 2024).

### 2.2 Executive functioning and generative AI

The EF refers to a set of cognitive processes, including working memory, inhibition, and cognitive flexibility (Diamond, 2013), which are essential for planning, concentration, and attention, and are crucial for academic performance in various ways. Although there are definitional disagreements regarding the exact components of EF, there appears to be a consensus that they are involved in goal-directed behavior requiring effortful problem-solving (Diamond, 2013; Gioia et al., 2015). In this study, we use the revised Behavior Rating Inventory of Executive Functioning (BRIEF-2; Gioia et al., 2015), a widely used EF rating scale, to measure various EFs, including inhibition, self-monitoring, flexibility, emotional control, task completion, working memory, and planning/organization.

In an educational setting, students rely on different cognitive processes for goal-directed behavior; for instance, when tackling writing assignments, they must independently initiate, plan, and complete tasks. Hence, EF plays a crucial role in successfully completing school assignments and achieving long-term academic success. Research has consistently linked lower EF to decreased academic achievement across various subjects, from childhood through adulthood (Best et al., 2011; Samuels et al., 2016; Pino Muñoz and Arán Filippetti, 2021). Preliminary evidence suggests that Swedish adolescents utilize generative AI tools as study aids for assignments that challenge their EF (The Nordic Youth Barometer, 2023).

Thus, the field of AI technology and its utilization among individuals with varying levels of EF is in a nascent stage. Until now studies on the intersection of AI and EF have primarily focused on exploring the potential of AI to assist clinicians in diagnosing various psychiatric conditions, including Attention Deficit Hyperactivity Disorder (ADHD) (Loh et al., 2022). In addition to this, certain studies have explored the potential of AI chatbots as supportive tools for children with special needs, such as Autism Spectrum Disorder (ASD) and ADHD, conditions which are frequently linked to lower EF (Tervo-Clemmens et al., 2023). This area appears to be in its preliminary stages of development as well (Torrado et al., 2023). While existing studies (e.g., Haleem et al., 2022) offer valuable insights into the impacts of various digital tools on the learning outcomes of young individuals, the introduction of new LLM AI chatbots since 2022 represents a significant advancement in the application of machine learning technologies in education. Their unparalleled accessibility and features signify a notable departure from traditional educational resources, including other digital tools, underscoring the urgent need for further research to comprehensively understand their implications. None of the aforementioned studies have investigated the new LLM AI chatbots and their effects on knowledge acquisition, and there is an apparent dearth of research focusing on non-clinical populations.

In an adult population, preliminary evidence suggests that use of ChatGPT can increase professionals’ productivity in writing tasks while maintaining the same quality of output, with participants with lower level of skills benefitting especially from their use (Noy and Zhang, 2023). A similar equalizing effect was observed in a different study (Brynjolfsson et al., 2023), where adult customer support agents with lower skill levels derived greater benefits from their use of the technology compared to their more highly skilled counterparts. Hence, it appears that adult users with lower skill levels seem to have larger relative gains from employing generative AI compared to their more skillful counterparts when solving tasks relevant to their work. It is uncertain if a comparable effect exists among adolescents when addressing tasks related to their academic studies. A different question is if a similar performance raising effect would be as desirable in an educational setting, where learning, not production, is the main goal. Might there be risks for decreases in learning? At the same time, research has indicated that children with poorer EF derive the greatest benefit from activities aimed to improve these functions (Diamond and Lee, 2011). Therefore, before implementing widespread structured use of generative AI in education, it is crucial to thoroughly examine the potential risks and benefits associated with its introduction.

While cheating by submitting work done by generative AI as one’s own is an obvious risk and a negative use of these tools, generative AI chatbots such as ChatGPT, if implemented safely, may also have a potential for strengthening individuals’ functioning in educational settings (Bai et al., 2023). In a recent study by Jauhiainen and Guerra (2023), ChatGPT demonstrated capabilities of customizing and personalizing learning material to match students at different levels. This was achieved by using ChatGPT to tailor the main text of lessons and attached exercises to three levels: basic, intermediate, and advanced. The level of each student was assessed by collecting grades in four key subjects (Jauhiainen and Guerra, 2023). In a recent quasi-experimental study on older adolescent students, the group with access to ChatGPT showed improvements compared to the non-user group in three subskills: knowing, applying, and reasoning. Additionally, qualitative insights revealed enhanced problem-solving in the experimental condition (Wardat and Alneyadi, 2024). Thus, students, particularly those with EF challenges, might benefit from using generative AI chatbots as aids in their schoolwork—for instance, in initiating or organizing academic tasks to facilitate timely completion of assignments. It can potentially provide an equalizing effect in terms of strengthening problem-solving skills in students with EF challenges for example by integrating AI-support into special education programs. Furthermore, generative AI could assist adolescents in various subjects and topics, providing external feedback on schoolwork and explaining concepts (Kasneci et al., 2023). However, relying excessively on AI chatbots as direct replacements for these functions, especially those involved in completing schoolwork, may diminish the very cognitive abilities they substitute (León Dominguez, 2024). Recent research emphasizes adolescence as a crucial stage for executive function development (Tervo-Clemmens et al., 2023), underscoring the importance of continuing to explore any tools that might either hinder or enhance the natural progression of EF during this life stage.

### 2.3 Present study

The present study had two primary objectives: (1) to investigate the frequency of usage and perceived usefulness of generative AI tools in schoolwork, including potential gender disparities in both usage and perceived usefulness; and (2) to examine how these usage patterns and perceived utility are associated with adolescents’ EF and academic achievement. These objectives were pursued through two separate studies. Study 1 focused on a sample of young adolescents, comprising middle school students, while Study 2 involved older adolescents, including high school students. Given the early stage of research on AI, particularly in educational settings, no specific hypotheses were posited, and the study was approached as an exploratory correlational investigation.

## 3 Study 1

### 3.1 Materials and methods

#### 3.1.1 Sample

The analytical sample of the study comprised 385 young adolescents (179 girls, 203 boys, 3 undisclosed or not identifying as either a girl or boy, 24.3% with foreign background).^2^ They were enrolled in seventh to ninth grade at four Swedish primary schools, with ages ranging from 12 to 16 years and a mean age of 14 years (*SD*_age_ = 0.85). These schools, located in the Southern part of Sweden, specifically in the Scania region with a population of over 1,340,000, shared similar socioeconomic status according to Statistics Sweden (SCB). All students in the relevant grades were invited to participate in the questionnaire, and the response rate was 80%. Additionally, all middle and high school students in Sweden receive laptops at no cost, regardless of whether they attend a municipal or private school.

#### 3.1.2 Measures

##### 3.1.2.1 The use of generative AI

Information regarding the utilization of generative AI was collected through items assessing its forms, frequency, and usefulness (three items), developed by the authors due to the lack of validated measures for the use of Generative AI for schoolwork. One of these items served as a gate question, permitting access to subsequent related inquiries only for those who responded affirmatively. The gate item was visible to all participants: *“I use AI services such as ChatGPT, My-AI on Snapchat, Youchat, Bing-chat, or Socratic in my schoolwork. For example, when doing homework or solving tasks in school.”* Participants responded with either ‘Yes’ or ‘No’.

Participants who answered ‘Yes’ to the gate item were directed to subsequent follow-up questions inquiring about the specific AI services they used, the frequency of their use of generative AI in schoolwork (on a 4-point scale from ‘rarely’ to ‘often’) and their agreement with three statements assessing the tools’ perceived usefulness for (1) initiating, (2) planning/organizing, and (3) completing schoolwork (rated on a 5-point scale from ‘not correct at all’ to ‘exactly right’). The scores from these three usability items for ChatGPT in schoolwork were aggregated to create a usability score, demonstrating high internal consistency with a Cronbach’s *α* value of 0.82.

##### 3.1.2.2 The BRIEF 2 self-report form

The revised Behavior Rating Inventory of Executive Functioning (BRIEF-2; Gioia et al., 2015) self report form was used to measure EF. The self-report form targets respondents aged 11–18 years and comprises 55 items divided into seven subscales: Inhibit (the ability to resist or not act on an behavioral impulse), Self-monitor (awareness of one’s impact on other people and outcomes), Shift (alteration of attention, flexibility in change, adjustment and problem-solving), Emotional control (the ability to regulate emotions), Task completion (the ability to complete tasks and/or homework on time), Working memory (the ability to hold information in mind during task completion) and Plan/organize (the ability to manage current- and future demands related to tasks). Summing the scores from the Inhibit and Self-monitor subscales composes the Behavioral Regulation Index (BRI), summing the scores from the Shift and Emotional control subscales composes the Emotional Regulation Index (ERI), and summing the scores from the Task completion, Working memory, and Plan/Organize subscales composes the Cognitive Regulation Index (CRI). Summing the scores of all indices collectively contributes to the Global Executive Composite (GEC), which serves as a global measurement of an individual’s EF. The BRIEF has demonstrated good validity and reliability across various countries (e.g., Pino Muñoz and Arán Filippetti, 2021; Huizinga et al., 2023). For instance, in the standardization sample for the BRIEF-2 self-report from, Cronbach alpha for the scales ranged between 0.81 and 0.97 (Gioia et al., 2015). In the present study, the BRIEF-2 demonstrated acceptable to excellent Cronbach’s alpha values, ranging from 0.71 for self-monitoring to 0.91 for task completion.

##### 3.1.2.3 Academic achievement

The participants’ grades in Swedish, Math, and English for the current academic year were collected from the schools and amalgamated to form an aggregate score representing their academic achievement. Grades in Swedish schools are denoted from F to A, with A being the highest. These grades were converted to a scale from 0 to 5 for each subject (Swedish, Math, and English) and were found to be highly correlated: Math correlated with Swedish at 0.70 and with English at 0.63, while the correlation between Swedish and English was 0.67 (all significant at *p* < 0.001). This demonstrates that the grades form a unidimensional construct with a Cronbach’s alpha of 0.85. The scores were then combined to create an overall measure of academic achievement.

#### 3.1.3 Procedure

This study was conducted as an exploratory correlation study and is part of the larger project titled “Well-being in School Environment,” led by Daiva Daukantaitė at the Department of Psychology, Lund University. Data collection took place at four secondary schools during a designated lecture hour, with both a teacher and a research assistant present. Each student received a personal link to a web-based survey, which they completed digitally using either personal or school-provided laptops. Prior to starting the survey, participants were briefed on the study’s purpose and content and were informed about the voluntary nature of their participation, providing consent accordingly. The survey took approximately 45 min to complete. In addition to the scales outlined in the preceding section, other measures related to mental health, emotional regulation, and life satisfaction were collected, although they were not utilized in this particular study. The research has been approved by the Swedish Ethics Committee (reference numbers: 2021–01666 and 2023–01013-02).

#### 3.1.4 Data cleaning

In total, 393 students participated in the survey. Eight participants who did not provide a response (either ‘yes’ or ‘no’) regarding their use of generative AI in schoolwork were excluded from the sample, resulting in a reduction from 393 to 385 participants.

Participants who responded “no” to the gate question about the use of generative AI in schoolwork were included in the analytic sample to investigate differences between users and non-users. Despite their response, these participants completed all questions regarding executive functions, allowing for a comparison of non-users with BRIEF-2 scores alongside the user group.

About 5% of participants had single missing items in the BRIEF-2. The handling of missing data adhered to the strict guidelines outlined in the BRIEF-2 manual (Gioia et al., 2015) during the data cleaning process.

#### 3.1.5 Statistical analysis

The statistical analyses were conducted using SPSS 29 (IBM Corp., 2017). Initial exploratory analyses examining gender and background differences among users and non-users of different generative AI tools for schoolwork were conducted using Chi-Square tests. Independent *t*-tests were employed to evaluate gender differences in the perceived usefulness of AI for schoolwork and differences in EFs between users and non-users of AI for schoolwork. Furthermore, Pearson correlations were performed to explore associations between measures of EFs, the frequency of use, perceived usefulness of generative AI for schoolwork, and academic achievement.

Assumptions regarding the homogeneity of group variances and normality of variables were assessed using Levene’s test for homogeneity of variance and Shapiro–Wilk tests, respectively. In instances where these assumptions were violated, Mann–Whitney *U* tests and Spearman’s correlations were utilized to validate the significance of the independent *t*-tests and Pearson correlations conducted.

Cohen’s *d* was employed as a measure of effect size for *t*-tests, where a value of 0.2 indicates a small effect size, 0.5 represents a medium effect size, and 0.8 signifies a large effect size (Cohen, 2013).

Given the exploratory nature of the study, numerous analyses were conducted, posing an increased risk for type 1 errors (false significance). To mitigate this risk, subscales for EF were only included in analyses if the Global Executive Composite, an overarching measure of EF problems, yielded significant results in preliminary analyses.

## 4 Results and discussion

### 4.1 Descriptive statistics

Out of 385 participants, 57 young adolescents (14.8%) indicated that they used some form of generative AI for schoolwork. These usage rates appear to be lower compared to findings among a youth sample aged 15–24 in Sweden (The Nordic Youth Barometer, 2023) and university students in Germany (von Garrel and Mayer, 2023), where approximately two-thirds reported using generative AI in their schoolwork.

Table 1 illustrates the types of generative AI tools utilized by participants in our study for their schoolwork; multiple choices were permitted. Among these tools, ChatGPT emerged as the most favored, being utilized by 40 (70%) adolescents. A Chi-Square test was conducted to examine whether the probability of using different generative AI tools for schoolwork varied by gender. As shown in Table 1, our analysis revealed that boys were more inclined to use ChatGPT for school-related tasks compared to girls, *χ^2^*_(1)_ = 3.96, *p* = 0.047, while no other notable gender differences were identified.

**Table 1:** Numbers of users of different generative AI-tools for schoolwork.

Type of tool	All		Girls		Boys		*χ^2^*	*p*
	*N*	%	*N*	%	*N*	%		
Any tool	57	100	24	100	31	100	0.27	0.612
ChatGPT	40	70	12	50	26	84	3.96	0.047
Bing-Chat	3	5.3	1	4.2	2	6.5		
YouChat	3	5.3	1	4.2	2	6.5		
Socratic	2	3.5	0	0	2	6.5		
Other^a^	25	44	14	58	11	35	0.92	0.341
Snapchat MY-AI^b^	20	35	12	50	8	26	1.46	0.233

^a^If Other was selected as choice, users were asked to specify which generative AI tool they used for schoolwork.^b^Snapchat MY-AI was the most common answer in the “Other” group.

A Chi-Square test assessed the likelihood of using generative AI tools for schoolwork based on foreign background. Results showed no significant differences, *χ^2^*_(1)_ = 0.11, *p* = 0.918.

### 4.2 Gender differences in frequency of use and perceived usefulness of generative AI for schoolwork

In terms of the frequency of use and perceived usefulness of generative AI for schoolwork among students who reported its usage, the majority indicated infrequent use, with 37.9% using it rather seldom and 46.6% using it very seldom, while only a small percentage reported using it very often (6.9%). These patterns did not differ significantly by gender, *χ^2^*_(3)_ = 1.14, *p* = 0.769.

Regarding perceived usefulness, Table 2 presents descriptive statistics and gender differences in perceived usefulness for schoolwork. While girls tended to rate the usefulness of generative AI for initiating, structuring, and completing various assignments slightly higher, no significant gender differences were observed.

**Table 2:** Gender differences in perceived usefulness of ChatGPT in initiation, structuring and completion of schoolwork.

Usefulness for schoolwork (ChatGPT)	Boys		Girls		Gender differences		
	*M*	*SD*	*M*	*SD*	*t*	*p*	Cohen’s *d*
Perceived usefulness for							
Initiation	4.13	1.36	4.42	1.38	0.59	0.560	0.21
Structuring	4.32	1.28	4.42	1.00	0.25	0.820	0.08
Completion	4.00	1.44	4.25	1.54	0.46	0.638	0.17
Overall usefulness	12.67	3.62	13.08	3.15	0.35	0.742	0.12

### 4.3 Differences between users and non-users of generative AI for schoolwork in self-rated EF

To investigate whether users of generative AI for schoolwork differed from non-users in self-rated EF, an independent *t*-test was initially conducted on BRIEF-2 self-report form Global Executive Component (GEC) scores. Users of generative AI for schoolwork reported significantly higher GEC scores, *t*(376) = 2.55, *p* = 0.011, Cohen’s *d* = 0.37, indicating that those who use AI for schoolwork reported significantly more EF problems. As depicted in Table 3, a notable difference was observed, particularly on the BRIEF-2 Emotional Control subscale, indicating that students with EF deficits may encounter difficulties in the self-regulatory skills required for initiating and completing tasks assigned by others.

**Table 3:** Differences in self-rated EF problems between students using- and not using generative AI for schoolwork.

Variable	Users (*N* = 57)		Non-users (*N* = 328)		Differences		
	*M*	*SD*	*M*	*SD*	*t*	*p*	Cohen’s *d*
Executive functioning							
Inhibit	14.75	4.07	13.53	3.56	2.34	0.020	0.34
Self-monitor	8.88	2.51	8.35	2.30	1.58	0.116	0.23
Shift	14.65	4.00	13.73	3.53	1.77	0.078	0.26
Emotional control	11.12	3.53	9.90	3.09	2.70	0.007	0.39
Task completion	13.74	4.21	12.72	3,79	1.84	0.067	0.26
Working memory	15.44	4.04	14.21	3.92	2.17	0.031	0.31
Plan/ organize	19.28	4.99	17.77	4.38	2.34	0.020	0.34
BRI	23.63	6.10	21.84	5.37	2.28	0.023	0.33
ERI	25.77	6.99	23.63	5.97	2.43	0.016	0.35
CRI	48.46	12.61	44.65	11.35	2.29	0.022	0.33
GEC	97.86	23.78	89.96	21.19	2.55	0.011	0.37

BRI, Behavior Regulation Index; ERI, Emotion Regulation Index; CRI, Cognitive Regulation Index; GEC, Global Executive Composite.

### 4.4 Relationships between frequency of use, perceived usefulness of generative AI for schoolwork and self-rated measures of EFs

The analysis exploring the relationships between the frequency of use, perceived usefulness of generative AI for schoolwork, and self-rated measures of EF was specifically conducted for the ChatGPT user group, given its sufficient sample size for analysis. The results revealed moderate positive significant correlations between the perceived usefulness of ChatGPT, the frequency of use, and EF problems (see Table 4). These findings suggest a potential compensatory relationship, wherein users with more EF problems reported finding ChatGPT more useful for initiating, structuring, and especially completing assignments in their schoolwork. This observation aligns with prior research indicating that individuals with lower skill levels may experience greater productivity gains from using ChatGPT compared to their more proficient counterparts (Brynjolfsson et al., 2023; Noy and Zhang, 2023).

**Table 4:** Pearson correlations between usefulness of ChatGPT for schoolwork, frequency of use of generative AI for schoolwork and global measure of EFs problems and academic achievement.

	Frequency of use	Global measure of EF problems (GEC)	Academic achievement
Perceived usefulness of ChatGPT for			
Initiation	0.48*	0.37*	0.02
Structuring	0.45*	0.28	0.19
Completion	0.36*	0.44*	−0.16
Overall usefulness	0.53*	0.41*	0.03

*p < 0.05. GEC, global executive composite.

### 4.5 Relationships between frequency of use and perceived usefulness of generative AI for schoolwork and academic achievement

We explored the associations between the frequency of use and perceived usefulness of generative AI for schoolwork and academic achievement. Although a modest trend suggested that higher grades were associated with finding ChatGPT useful for structuring (*r* = 0.19), conversely, there was a slight inclination for lower grades to be linked with finding the tools helpful for completing school assignments (*r* = −0.16). However, these correlations, including those with frequency of use (*r* = −0.05) and finding tools useful for initiating (*r* = 0.02), were generally weak and not statistically significant (see Table 4).

## 5 Study 2

To broaden the applicability of our findings from Study 1, we explored the frequency and perceived usefulness of AI chatbots for schoolwork, as well as their association with EF problems, among older adolescents in high school (aged 15–19) in Study 2. Expanding on our insights garnered from schools and recent studies published subsequent to our data collection in Study 1, we incorporated additional dimensions of usefulness based on findings from German and Swedish research (The Nordic Youth Barometer, 2023; von Garrel and Mayer, 2023), as outlined in the Measures section. Additionally, we expanded our assessment by including teacher ratings of adolescents’ EF alongside self-rated EF, aiming to compare results obtained from both sources. However, measures of academic achievement were not available for Study 2, as this data for high school students typically becomes accessible only at the end of the school year.

### 5.1 Materials and methods

#### 5.1.1 Sample

The sample comprised 359 adolescents (239 girls, 111 boys, 9 undisclosed or not identifying as either a girl or boy; 14.8% with foreign background). They were enrolled in the first to third year at one large Swedish high school (gymnasium) with approximately 1,200 students and 5 different main programs. The participants’ ages ranged from 15 to 19 years with a mean age of 17 years (*SD_age_* = 0.89) representing the various main programs. All students from the selected programs were invited to participate in our survey, resulting in an 81% response rate.

The school is a communal one with relatively high entrance requirements, drawing students from across the Scania region. The region has a unique arrangement between municipalities, allowing students to freely choose their schooling from any part of the larger region, whether private or municipality schools, without tuition fees. As previously mentioned, all high school students in Sweden receive laptops at no cost.

#### 5.1.2 Measures

##### 5.1.2.1 The use of generative AI

The same measure employed in Study 1 to assess the use of generative AI was utilized, with additional items included to evaluate the perceived usefulness of generative AI for schoolwork. In addition to rating the perceived usefulness for initiation, structuring, and task completion, we introduced items regarding perceived usefulness for summarizing, improving texts, explaining concepts, and writing texts. These four additional items were worded similarly to the original questions from Study 1, prompting participants to indicate their level of agreement with each statement on a 5-point scale ranging from ‘not correct at all’ to ‘exactly right.’ The Cronbach’s alpha for the extended measure for usability of generative AI in schoolwork was 0.74.

Furthermore, we incorporated one item linked to the use of generative AI and avoidance of effortful thinking in schoolwork, which was worded as follows: “I prefer to ask an AI tool for help rather than try on my own when I encounter difficulties in my schoolwork” (rated on a 5-point scale from ‘not correct at all’ to ‘exactly right’). This item will be referred to as ‘Avoiding effort’ in the forthcoming results section, which will follow the structure of the results section for Study 1.

##### 5.1.2.2 The BRIEF-2 self-report

The BRIEF-2 self-report form, described in more detail in Study 1, was used to measure EF in Study 2 as well. In this study, the BRIEF-2 demonstrated acceptable to excellent Cronbach’s alpha values, ranging from 0.73 for self-monitoring to 0.90 for task completion.

##### 5.1.2.3 The BRIEF-2 teacher-report

In addition to the Self-report form, we also utilized the BRIEF-2 Teacher-report form in this study, which is designed for assessing EF in children and adolescents aged 5–18. The teacher form consists of 63 items with nine scales of EF. Seven of these scales (Inhibit, Self-Monitor, Shift, Emotional Control, Working Memory, and Plan/Organize) correspond to the scales in the self-report version. Additionally, two new scales are introduced: Task-Monitor (which assesses difficulties in recognizing minor errors in work output) and Organization of Materials (evaluating the orderliness of workspaces, play areas, and storage spaces) (Gioia et al., 2015). Summing the scores from the nine scales of the teacher form comprise three indices: BRI, ERI, and CRI, as well as the overall score GEC (Gioia et al., 2015). As documented in the BRIEF-2 manual (Gioia et al., 2015), the test–retest reliability for the teacher form was 0.87, and the Cronbach’s alpha values for the scales and indices were excellent, ranging from 0.81 to 0.97. In this study, the BRIEF-2 demonstrated excellent Cronbach’s alpha values, ranging from 0.86 for organization of materials to 0.95 for emotional control.

#### 5.1.3 Procedure

Data was collected at a large high school. Similar to Study 1, each student received a personalized link to a web-based survey, which they completed digitally using either personal or school-provided laptops. Participants received an information letter outlining the project’s aims and their right to withdraw at any time without needing to provide reasons, along with contact information for the project leader in case they had additional questions via email, the week before data collection. Before completing the survey, students were asked to provide consent accordingly. The survey took approximately 30 min to complete. The research has been approved by the Swedish Ethics Committee (reference numbers: 2021–01666 and 2023–01013-02).

#### 5.1.4 Data cleaning

In total, 359 students participated in the survey, and all of them responded to a question regarding their use of generative AI in schoolwork, with options ranging from “never” to “very often.” Participants who responded “never” to the gate question about the use of generative AI in schoolwork were included in the analytic sample to investigate differences between users and non-users and also completed all questions regarding executive functions, allowing for a comparison of non-users with BRIEF-2 scores alongside the user group.

About 4% of participants had single missing items in the BRIEF-2. The handling of missing data adhered to the strict guidelines outlined in the BRIEF-2 manual (Gioia et al., 2015) during the data cleaning process.

#### 5.1.5 Statistical analysis

As in Study 1, statistical analyses were conducted using SPSS 29 (IBM Corp., 2017). Chi-Square tests examined gender and background differences among AI tool users and non-users. Independent t-tests assessed gender differences in perceived AI usefulness and EFs differences. Pearson correlations explored associations between EFs, AI use and perceived usefulness.

Assumptions of variance homogeneity and normality were checked with Levene’s and Shapiro–Wilk tests. Violations were addressed with Mann–Whitney *U* tests and Spearman’s correlations. Cohen’s *d* measured effect sizes for *t*-tests (0.2: small, 0.5: medium, 0.8: large).

To reduce type 1 errors, EF subscales were analyzed only if the Global Executive Composite showed significant results.

## 6 Results study 2 and discussion

### 6.1 Descriptive statistics

Out of 357 participants, 189 adolescents (52.6%) indicated that they used some form of generative AI for schoolwork, a percentage that was still lower than those found in published Swedish (The Nordic Youth Barometer, 2023) and German studies (von Garrel and Mayer, 2023). Table 5 presents which types of generative AI tools they use in their schoolwork; multiple choices were allowed. ChatGPT was the most popular generative AI for schoolwork, used by 168 (88.9%) adolescents.

**Table 5:** Numbers of users of different generative AI-tools for schoolwork.

Type of tool	All		Girls		Boys		*χ^2^*	*p*
	*N*	%	*N*	%	*N*	%		
Any tool	189	100	126	100	61	100	3.17	0.574
ChatGPT	168	88.9	106	84	60	98	8.04	0.004
Bing-Chat	4	2.1	2	1.6	2	3.3		
Bard	3	1.6	1	0.7	2	3.3		
Snapchat MY-AI	61	100	51	40	9	15	12.48	<0.001
Other	7	3.7	6	4.7	1	1.6		

A Chi-Square test was conducted to determine whether the likelihood of being a user of different generative AI tools for schoolwork differed based on gender. As presented in Table 5, we found that boys were more likely to use ChatGPT, *χ^2^*_(1)_ = 8.04, *p* = 0.004, while girls preferred Snapchat MY-AI for schoolwork, *χ^2^*_(1)_ = 12.48, *p* < 0.001.

A Chi-Square test assessed the likelihood of using generative AI tools for schoolwork based on foreign background. A trend indicated that participants with a foreign background were somewhat more likely to use generative AI, though this was not statistically significant, *χ^2^*(1) = 3.78, *p* = 0.052.

### 6.2 Gender differences in frequency of use and perceived usefulness of generative AI for schoolwork

Regarding the frequency of generative AI use for schoolwork among students who reported using AI, the majority used it rather seldom (50%) or sometimes (28%), while a smaller percentage used it rather often (17.4%) and often (4.2%). No gender differences were observed in these patterns, *χ*^2^_(3)_ = 1.09, *p* = 0.780.

As for perceived usefulness, Table 6 shows that no significant gender differences were found in the perceived usefulness of generative AI for schoolwork.

**Table 6:** Gender differences in perceived usefulness of generative AI in schoolwork and for avoiding effort.

	Boys		Girls		Gender differences		
	*M*	*SD*	*M*	*SD*	*t*	*p*	Cohen’s *d*
Perceived usefulness for							
Initiation	3.32	1.37	3.13	1.36	−0.91	0.365	−0.14
Structuring	2.80	1.18	2.44	1.32	−1.85	0.066	−0.28
Completion	2.28	1.2	2.57	1.32	1.44	0.153	0.23
Summarization	3.77	1.13	3.69	1.20	−0.4	0.693	−0.06
Improving texts	2.45	1.27	2.46	1.30	0.05	0.962	0.01
Explaining concepts	3.38	1.35	3.7	1.44	1.42	0.156	0.22
Writing texts	2.37	1.27	2.21	1.29	−0.82	0.413	−0.13
Overall usefulness	20.41	4.96	20.19	5.34	−0.27	0.787	−0.04
Avoiding effort	2.02	1.03	2.21	1.08	1.13	0.259	0.18

### 6.3 Differences between users and non-users of generative AI for schoolwork in self- and teacher-rated EFs

To investigate whether users of generative AI for schoolwork differed from non-users in self-rated and teacher-rated EF, two independent *t*-tests were conducted. Although both self-reported (*M* = 94.31, *SD* = 19.66) and teacher-reported EF problems (*M* = 71.00, *SD* = 13.85) were somewhat higher among users compared to non-users (*M* = 91.26/69.15, *SD* = 18.45/13.93 for self/teacher ratings), no significant differences were found between the two groups in either self-reported or teacher-rated EF problems, as measured with the BRIEF-2 Global Executive Component (GEC).

### 6.4 Relationships between frequency of use, perceived usefulness of generative AI for schoolwork and self-and teacher-rated EFs

To explore the relationships between the frequency of use and perceived usefulness of generative AI for schoolwork and self- and teacher-rated EF problems, Pearson correlations were conducted. The frequency of use of generative AI for schoolwork showed positive and significant correlations with all aspects of perceived usefulness. Regarding EF problems, the most prominent and moderately strong correlations were observed between self- and teacher-rated EF and perceived usefulness for completing schoolwork (*r* = 0.29, *p* < 0.01 for both self and teacher ratings), followed by perceived usefulness for improving texts (*r* = 0.18 and 0.17, *p* < 0.05) and effort avoidance (*r* = 0.21 and 0.19, *p* < 0.05) (see Table 7). For informational purposes, the overall scores of self- and teacher-rated EFs were moderately positively correlated (*r* = 0.39, *p* < 0.001).

**Table 7:** Pearson correlations between perceived usefulness of generative AI for schoolwork and frequency of use of generative AI for schoolwork and measures of EF.

		Global measure of EF problems (GEC)	
	Frequency of use	Self-rated	Teacher-rated
Perceived usefulness for			
Initiation	0.37***	0.01	0.01
Structuring	0.39***	0.10	0.02
Completion	0.38***	0.29***	0.29**
Summarizing	0.28***	−0.07	0.04
Improving texts	0.26***	0.18*	0.17*
Explaining concepts	0.19*	0.11	0.00
Writing texts	0.25***	0.19*	0.10
Overall usefulness	0.52***	0.26***	0.14
Effort avoidance	0.50***	0.21**	0.19*

*p < 0.05, **p < 0.01, ***p < 0.001.GEC, global executive composite.

## 7 General discussion

In this study, our primary aim was to investigate the frequency and perceived usefulness of LLM generative AI chatbots for schoolwork, focusing specifically on their relationships with adolescents’ executive functioning (EF), which encompasses cognitive processes, including planning, inhibition, and cognitive flexibility, all critical for academic success. To accomplish this aim and ensure the validity of the findings we conducted two studies involving both younger and older adolescents, aiming to comprehensively examine these relationships across different age groups. As far as we are aware, this study represents the first attempt to explore these aspects among adolescents, providing valuable insights into their use of LLM generative AI chatbots and their implications for academic performance and cognitive development.

### 7.1 Frequency of use of generative AI in schoolwork

In Study 1, approximately 14.8% of participants reported using generative AI, while in Study 2, this figure was 52.6%. The notable disparity in usage rates between the two studies may be attributed to several factors. Firstly, the temporal aspect likely played a role, with Study 2 conducted nearly a year later than Study 1, suggesting a potential rise in the adoption of generative AI over time. Additionally, differences in participant age between the two studies could have influenced usage rates, with older adolescents possibly demonstrating a higher inclination to utilize such technology. This is supported by findings from other studies, such as The Nordic Youth Barometer (2023) and research by von Garrel and Mayer (2023), which also indicate an upward trajectory in generative AI usage with age. Moreover, as older adolescents often encounter more complex assignments in their schoolwork, they might rely more on generative AI tools to aid them. Lastly, variations in school environments, such as differences in technological infrastructure or educational practices, might have contributed to the observed differences in usage rates.

Although our findings reveal variability in the adoption of generative AI among different adolescent populations, the usage rates observed in our studies were notably lower compared to those reported in studies involving older youth populations, such as university students in Germany (von Garrel and Mayer, 2023) and a broader age range of youths (15–24 years) in Sweden (The Nordic Youth Barometer, 2023), where usage rates reached as high as two-thirds. One potential explanation for this disparity, as mentioned above, is the age difference among participants. Additionally, variations in the formulation of our survey questions, albeit slight, might have influenced participant responses. Finally, while data collection in previously published studies likely occurred anonymously and remotely, in our study we collected data in school settings using pseudoanonymization (assigning each participant a numerical code, with their names registered on a single master list). Despite assuring participants that their information would be treated confidentially and that individual data would not be analyzed or presented, this approach may have led to underreporting of usage rates. Therefore, further research involving data collection in school settings and utilizing diverse methodologies is warranted to delve deeper into this phenomenon.

### 7.2 The perceived usefulness of generative AI in schoolwork and its relationship to executive functioning and academic achievement

Only in Study 1 did we observe that users of AI for schoolwork reported significantly more EF problems compared to non-users. However, in both studies, we found a consistent pattern indicating that users of generative AI in schoolwork with more EF problems perceived AI as more useful for schoolwork, particularly regarding its perceived usefulness for completing school-related assignments. This result may be linked to previous research indicating that individuals with lower work-related skill levels (e.g., difficulties in writing tasks relevant to their work) derive greater productivity improvements from using AI tools compared to their more proficient counterparts (Brynjolfsson et al., 2023; Noy and Zhang, 2023). However, our findings also raise important questions about the manner in which AI is utilized in completing school assignments. Specifically, it remains unclear whether AI is primarily used to assist with tasks already initiated or to independently complete entire assignments. This distinction is crucial as it not only informs discussions surrounding the ethical implications of AI usage in education but also highlights concerns about academic integrity and the uncritical reliance on AI-generated material. For instance, Noy and Zhang (2023) found that a majority of individuals who reported productivity gains simply copied and pasted output from ChatGPT. If AI is used uncritically, with a reliance on AI-generated material, it poses risks beyond academic integrity. Such risks include potential biases in the output (Bender et al., 2021) and the dissemination of outdated or harmful content, as evidenced by the discovery of race-based content in healthcare applications (Omiye et al., 2023). Therefore, it is imperative to address not only the ethical concerns but also the broader implications of uncritical AI usage in academic settings.

In our study, we observed that adolescents with more EF problems predominantly use ChatGPT and other generative AI tools for completing assignments rather than for initiating or structuring them, which raises significant concerns. While utilizing these tools for initiation or structuring, especially in the initial stages of structured AI usage in a school setting, could potentially enhance or augment existing abilities and foster educational attainment, their direct substitution—where these tools replace rather than enhance existing abilities—may exacerbate academic disparities in the long term. Recent research underscores adolescence as a pivotal period for EF maturation (Tervo-Clemmens et al., 2023), emphasizing the necessity of continued investigation into any tools that may interfere with or amplify the natural development of EFs during this life stage. Additionally, León-Domínguez (2024) proposes theoretical scenarios, informed by neuroscience, to explore the potential outcomes of increased generative AI usage among adolescents. A primary concern is the possibility of certain groups developing an excessive dependence on these technologies, which might encourage the evasion of challenging cognitive tasks, potentially resulting in a stagnation or deterioration of cognitive capabilities in the long term (León-Domínguez, 2024). This highlights the importance of considering the broader implications of generative AI usage among adolescents and the need for proactive measures to mitigate potential negative impacts on cognitive development and academic achievement.

### 7.3 Strengths and limitations

The study has several strengths, including the utilization of two relatively large samples of adolescents and a high response rate, which enhances the study’s external validity by providing a diverse representation. Moreover, the inclusion of teacher ratings of EF in Study 2 adds depth and validation to the results, offering a more comprehensive understanding of the relationship between generative AI use and EF among adolescents.

However, there are also some limitations to consider. Firstly, the survey for Study 1 was conducted in spring 2023, during the early stages of introducing generative AI chatbots. Consequently, the results may not reflect current trends or usage patterns, as technology adoption and usage habits may have evolved since then and the timing of the study could be a potential confound. Furthermore, more nuanced research is needed to clarify how AI chatbots were used for different school-related tasks, such as whether they assisted with tasks already initiated by students or were used to independently complete entire assignments. Understanding these distinctions could provide deeper insights into the specific ways AI chatbots are utilized. Additionally, the relatively low number of AI users for schoolwork observed in Study 1 may have impacted the statistical power of our analyses, potentially leading to Type II errors.

Another limitation lies in the reliance solely on self-report measures for EF in Study 1. To overcome this, future research could integrate teacher and parent ratings for younger individuals and explore performance-based assessments in schoolwork with and without generative AI. This approach would offer a more comprehensive understanding of the impact of these tools on EF among adolescents.

Additionally, the absence of validated scales for evaluating the usefulness of ChatGPT and other generative AI tools in schoolwork poses a challenge. Custom questions and scales were developed for this study, but they may not fully capture the relevant factors. While the scale for the usefulness of ChatGPT demonstrated good internal consistency in both studies, further validation in future research is necessary Moreover, a more precise inquiry—such as quantifying how frequently students use AI tools for schoolwork in terms of times per week or day—might yield more insightful results compared to the more ambiguous response options like “rarely” or “often” used in the current study.

Furthermore, in Study 2, data was collected from a single, albeit large, school, which may introduce selection bias. Therefore, conducting multi-site studies would enhance the generalizability of the results. However, the classes from the school were chosen randomly, covering a broad range of specialties, and the response rate of 81% may provide a good representation of that age group of adolescents. While non-significant differences regarding foreign background were found in both studies, a clear tendency observed in Study 2 suggests that this should be studied further in a larger sample to examine the nuances of these relationships as well as their connections to EF. Given the novelty of this research field, further exploration of other potential covariates related to the relationships between AI use, perceived usefulness, and EF/academic achievement would be beneficial.

Lastly, the extensive number of analyses conducted in this study increases the risk of type 1 errors, which should be taken into account when interpreting the findings. Given the exploratory nature of the research and the limited existing literature on the topic, further validation through additional research studies is warranted.

### 7.4 Practical and theoretical implications

The utilization of generative AI for schoolwork appears to be relatively uncommon among Swedish youths aged 12–16, as evidenced by the findings of Study 1. However, in a slightly older population, comprising high school students, more than half reported using these tools for their academic tasks. Although the discrepancy could be partially attributed to age differences, in line with current research (The Nordic Youth Barometer, 2023; von Garrel and Mayer, 2023), the almost one-year difference between data collections is likely to be a significant confound. The results may not fully reflect current trends or usage patterns, given that technology adoption and usage habits could have evolved since the data were collected. Nevertheless, the introduction of such tools, with the potential to amplify, impact, or substitute critical abilities necessary for academic success, without thorough scientific investigation, raises significant concerns.

Educators should take note of the implications highlighted by this study, particularly regarding the potential risks associated with generative AI use among students with EF difficulties. Evidence from both Study 1 and Study 2 suggests that these individuals may be more inclined to use AI tools and may prefer utilizing them in a manner that does not enhance existing abilities. This could have negative implications for their long-term learning outcomes and the natural development of EF.

However, if thoughtfully implemented, AI chatbots might have the potential to aid students, especially those with EF deficits, by enhancing their ability to plan and manage tasks effectively. Given the link between EF deficits and academic struggles, which can lead to a vicious cycle resulting in early school dropout (Esch et al., 2014), these tools could potentially bridge the gap between students with varying levels of EF, offering opportunities for educational equity.

## 8 Conclusion

This study represents the first exploration of how individual characteristics, such as EF, relate to the frequency and perceived usefulness of LLM generative AI chatbots for schoolwork among adolescents. By conducting two studies involving both younger and older adolescents, we gained valuable insights into these relationships across different age groups. Our findings illuminate the usage patterns of generative AI among the studied adolescents, although it is important to note that these patterns could have evolved since the data were collected. The observed disparities between studies underscore the necessity for further investigation into the factors influencing generative AI usage rates among adolescent populations. Future studies should also evaluate whether there are preferences for different tools tied to gender, as the use of various tools may impact users in different ways. The potential gender-based differences in the likelihood of using tools like ChatGPT and Snapchat MY-AI for schoolwork could be a significant topic for future research. If these tools demonstrate varying effectiveness when used for schoolwork, differences in usage between genders could have negative implications from an equality standpoint.

Our findings reveal that adolescents with more EF problems tend to perceive generative AI tools as more useful for schoolwork, especially for completing assignments. This association prompts questions about the role of AI in education and its potential impact on academic integrity and ethical considerations.

This study emphasizes the urgency for policymakers, researchers, and educators to carefully evaluate the integration of generative AI into school environments. While our findings suggest potential risks associated with generative AI use, particularly among students with EF difficulties, these tools also may offer the possibility of aiding students in managing academic tasks more effectively. Further research and proactive measures are essential to ensure the safe and effective use of these technologies, while also considering their implications for academic equity and cognitive development among adolescents.

## Data availability statement

The raw data supporting the conclusions of this article will be made available by the authors, without undue reservation.

## Ethics statement

The studies involving humans were approved by Swedish Ethics Committee (reference numbers: 2021–01666 and 2023–01013-02). The studies were conducted in accordance with the local legislation and institutional requirements. The ethics committee/institutional review board waived the requirement of written informed consent for participation from the participants or the participants’ legal guardians/next of kin because they deemed that passive informed consent from the participants’ legal guardians/next of kin was sufficient to mitigate the risk of selection bias. However, digital informed consent was obtained from all participants.

## Author contributions

JK: Conceptualization, Formal analysis, Investigation, Writing – original draft. EH: Conceptualization, Methodology, Supervision, Writing – review & editing. AL: Writing – review & editing. DD: Conceptualization, Data curation, Funding acquisition, Methodology, Project administration, Supervision, Writing – review & editing.

## Footnotes

^1^ https://info.ungdomsbarometern.se/publika-rapporter/back2school-2023

^2^Defined as the child either being born abroad with at least one parent born abroad as well or being born in Sweden with both parents being born abroad.
