# Supplementary material for: Adolescents’ use and perceived usefulness of generative AI for schoolwork: exploring their relationships with executive functioning and academic achievement
Source: Front Artif Intell. 2024 Aug 28;7:1415782. doi: 10.3389/frai.2024.1415782 (PMC11387220; doi:10.3389/frai.2024.1415782)
Supplement: Supplementary file 1 [file Table_1.docx]

Supplementary materials

The following items were originally distributed to participants in Swedish and have been translated into English for reporting purposes.

**Table S1**

*Questions Regarding AI Use in Schoolwork Asked in Study 1*

| 1. **Do you utilize AI chatbots such as ChatGPT, Bing chat, Youchat, Socratic, or other in your schoolwork, such as for completing homework or solving assignments in school?**   ❑1 Yes ❑2 No  [If participants selected "Yes," they proceeded to the following questions. If "No," they were redirected to other parts of the survey.] |
| --- |
| 1. **Which AI chatbot do you use? (**You can choose multiple options**)**   ❑1 ChatGPT  ❑2 Bing Chat  ❑3 Youchat  ❑4 Socratic  ❑5 Other  If 'Other', please specify which AI chatbots do you use?  …………………………………………………….. |
| 1. **How often do you use AI chatbots in your schoolwork?**   ❑_1_ Rarely ❑_2_ Sometimes ❑_3_ Often ❑_4_ Very often |

|  | Strongly Disagree | Disagree | Neither agree nor disagree | Agree | Strongly Agree |
| --- | --- | --- | --- | --- | --- |
| 1. **The AI tools that I use help me to...** |  |  |  |  |  |
| 1. ...get started with assignments in my schoolwork. | _1_ | _2_ | _3_ | _4_ | _5_ |
| 1. ...structure and organize when I work with assignments in my schoolwork. | _1_ | _2_ | _3_ | _4_ | _5_ |
| 1. ...complete assignments in my schoolwork. | _1_ | _2_ | _3_ | _4_ | _5_ |

**Table S2**

*Questions Regarding AI Use in Schoolwork Asked in Study 2*

| 1. **How often do you use some kind of AI tool in your schoolwork, for example, ChatGPT, Bing-chat, MY-AI on Snapchat, or Google bard when doing homework or solving assignments in school?**   ❑_0_ Never ❑_1_ Rarely ❑_2_ Sometimes ❑_3_ Often ❑_4_ Very often  [If participants selected "Rarely" to "Very often," they proceeded to the following questions. If they selected "Never," they were redirected to other parts of the survey.] |
| --- |
| 1. **Which chatbots do you use? (**You can choose multiple options**)**   ❑1 ChatGPT  ❑2 Bing Chat  ❑3 My-AI on Snapchat  ❑4 Google bard  ❑5 Other  If 'Other', please specify which chatbots do you use?  …………………………………………………………………………………………………………………………………………………………………………………………………………….... |

|  | Strongly Disagree | Disagree | Neither agree nor disagree | Agree | Strongly Agree |
| --- | --- | --- | --- | --- | --- |
| 1. **The AI tools that I use help me to...** |  |  |  |  |  |
| 1. ...get started with assignments in my schoolwork. | _1_ | _2_ | _3_ | _4_ | _5_ |
| 1. ...structure and organize when I work with assignments in my schoolwork. | _1_ | _2_ | _3_ | _4_ | _5_ |
| 1. ...complete assignments in my schoolwork. | _1_ | _2_ | _3_ | _4_ | _5_ |
| 1. …summarize knowledge. | _1_ | _2_ | _3_ | _4_ | _5_ |
| 1. …improve texts. | _1_ | _2_ | _3_ | _4_ | _5_ |
| 1. …...explain and clarify concepts that I don’t understand. | _1_ | _2_ | _3_ | _4_ | _5_ |
| 1. ...write texts | _1_ | _2_ | _3_ | _4_ | _5_ |
| 1. If you use AI tools for assistance with anything other than the above in your schoolwork, please describe it here …………………………………………………………………….   …………………………………………………………………………………………….. | | | | | |

| 1. **I’d rather ask an AI tool for help than try myself when I encounter difficulties in my schoolwork.**   ❑1 Strongly disagree  ❑2 Disagree  ❑3 Neither disagree nor agree  ❑4 Agree  ❑5 Strongly agree |
| --- |
