# Supplementary figures and images for: Adolescents’ use and perceived usefulness of generative AI for schoolwork: exploring their relationships with executive functioning and academic achievement
Source: Front Artif Intell. 2024 Aug 28;7:1415782. doi: 10.3389/frai.2024.1415782 (PMC11387220; doi:10.3389/frai.2024.1415782)

**Figure S1**

*Distribution of Global Executive Composite Scores in Sample 1 (a) and Sample 2 (b)*

(a)


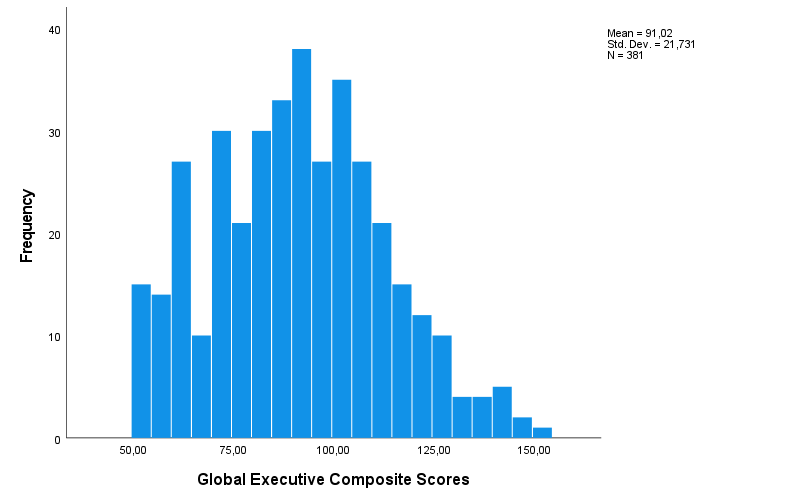


(b)


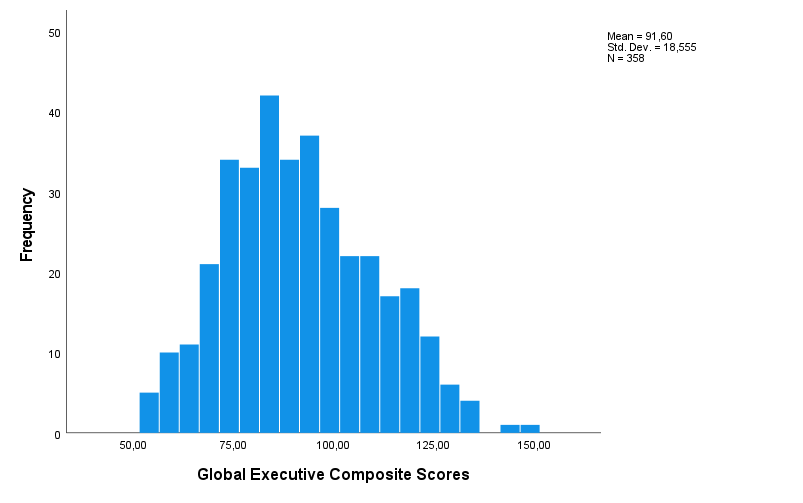

Supplement: Supplementary file 2 [file Data_Sheet_1.docx]
